# Predicting Disparity between ASF-Managed Areas and Wild Boar Habitats: A Case of South Korea

**DOI:** 10.3390/ani13223482

**Published:** 2023-11-11

**Authors:** Chanwoo Ko, Dongwook W. Ko, Wonhee Cho

**Affiliations:** 1 Department of Forest Resources, Kookmin University, 77 Jeongneung-ro, Seongbuk-gu, Seoul 02707, Republic of Korea; kocw0503@kookmin.ac.kr; 2 Department of Forest, Environment, and Systems, Kookmin University, 77 Jeongneung-ro, Seongbuk-gu, Seoul 02707, Republic of Korea; dwko@kookmin.ac.kr; 3 Industry Academic Cooperation Foundation, Kookmin University, 77 Jeongneung-ro, Seongbuk-gu, Seoul 02707, Republic of Korea

**Keywords:** ASF, SDM, potential risk areas, disease management strategies, transmission pathways

## Abstract

**Simple Summary:**

African swine fever (ASF) is a highly contagious viral disease that affects domestic pigs and wild boars worldwide. For efficient and accurate ASF management, identification of potential wild boar habitats and current ASF-managed areas is crucial. In this study, we used ensemble species distribution models to estimate the potential habitats of wild boar and ASF-managed areas. We aimed to identify areas of disparity between wild boar habitats and managed areas to identify potential risk areas for ASF transmission. Additionally, we expect that this study will ensure effective management.

**Abstract:**

African swine fever (ASF) is a highly contagious viral disease affecting both domestic and wild boars. Since its first outbreak in South Korea in 2019, substantial efforts have been made to prevent ASF transmission by reducing the wild boar population and eliminating infected carcasses; however, the persistence of ASF transmission has posed challenges to these efforts. To improve ASF management strategies, the limitations of current management strategies must be identified by considering disparities between wild boar habitats and ASF-managed areas with environmental and anthropogenic characteristics of wild boars and their management strategies. Here, ensemble species distribution models were used to estimate wild boar habitats and potential ASF-managed areas, with elevation, distance to urban areas, and Normalized Difference Vegetation Index as important variables. Binary maps of wild boar habitats and potential ASF-managed areas were generated using the maxSSS as the threshold criterion. Disparity areas of ASF management were identified by overlying regions evaluated as wild boar habitats with those not classified as ASF-managed areas. Dense forests near urban regions like Chungcheongbuk-do, Gyeongsangbuk-do, and Gyeongsangnam-do were evaluated as disparity areas having high risk of ASF transmission. These findings hold significant potential for refining ASF management strategies and establishing proactive control measures.

## 1. Introduction

African swine fever (ASF) is a highly contagious viral disease affecting both domestic pigs and wild boars. The disease originated in sub-Saharan Africa and was first described in Kenya in 1921 [1]. The first global expansion of ASF occurred across Europe after 1957, when the virus was introduced in Portugal [1,2]. Since its first spread in Europe, it has continuously spread across Europe, South America, and Asia because of increased globalization resulting in international movement of people and products [3].

ASF is primarily transmitted via direct contact with infected pigs, body fluids, tissues, and excrements [4,5,6]. Wild boars play a crucial role as both hosts and vectors in the spread and retention of the ASF virus in natural ecosystems. Wild boar behaviors, such as sound mobility, home range expansion, and omnivorous and scavenging foraging, have led to the rapid spread of ASF to new regions and challenges in ASF control [7]. In addition, some individuals in Europe have survived ASF infection without showing signs of the disease [8]. These asymptomatic wild boars can become persistently infected with the ASF virus and shed it in their body fluids, feces, and secretions over long periods [9,10,11]. 

ASF poses a significant threat to human benefits by damaging the global pig industry and food security [3,12,13,14]. It still remains an outbreak in European countries, with 1.3 million pigs being lost to ASF between 2016 and 2020 [15]. ASF outbreaks have occurred in China, Vietnam, India, Indonesia, Philippines, North Korea, and South Korea. To prevent further spread of ASF, culling (or hunting) of wild boars has been conducted in many countries, leading to a decrease in pork production, an increase in the price of pork-based goods, and vulnerability of regions where pork is a staple food [16,17,18]. Understanding and managing the spread of ASF is vital for sustaining local and global economics, food security, biosecurity, and the environment.

Since the first outbreak in Europe, human society has made efforts to prevent the spread of ASF. Various management strategies have been applied to control the spread of ASF, such as wild boar hunting, elimination of infected carcasses, and installation of artificial and natural barriers to prohibit its movement [19,20,21]. Wild boar hunting has the advantage of reducing the population density of wild boars, making it more difficult for the virus to spread among the remaining population. South Korea has invested significant efforts in hunting wild boars, especially in the Gyeonggi-do and Gangwon-do municipalities, where the number of wild boars were estimated to decline from 2.1 to 0.19 individuals per km^2^ by hunting from 2019 to 2021 [22]. Unfortunately, the virus has continued to spread to new municipalities. 

Human society has undertaken substantial efforts to prevent ASF transmission by reducing the wild boar population and eliminating infected carcasses in natural ecosystems. However, the ongoing challenges due to the persistence of ASF transmission underscore the inherent limitations of current management strategies. In particular, knowledge and operational disparities exist between the natural core habitats of wild boars and the designated ASF-management areas [23]. Considering the ecological-anthropogenic characteristics of wild boars and current ASF management strategies, it is important to reduce these management disparities.

Species distribution models (SDMs) are widely used to predict environmental niches, core habitats, and the probability of distribution of target species across landscapes [24,25,26,27]. To map the distribution probability of the target species, spatial information on the occurrence points and environmental variables, which represent topography, anthropogenic factors, and climate, has been used to estimate the correlation between species occurrence and environmental factors [24,28,29]. Many algorithms, such as Maximum Entropy (MaxEnt), Random Forest (RF), General Additive Models (GAMs), support vector machines (SVM), and boost regression trees (BRT) have been developed and applied in previous studies [30,31,32]. Furthermore, ensemble algorithms have also been developed to increase prediction accuracy [33].

Many previous ASF studies have applied SDMs to predict wild boar habitats to support the management of ASF spread [16,30,34,35,36]. Choi et al. (2023) [16] estimated the shortest paths for an ASF outbreak by using MaxEnt and network analysis. Bergmann et al. (2021) [37] estimated wild boar habitats in the European continent and applied them to determine the areas for installing barriers that prevent wild boar movement. Ko and Cho et al. (2023) [38] developed a model to assess the effect of ASF management strategies based on an agent-based model that can assess the efficiency of hunting intensity using wild boar habitat predictions. However, these studies have limitations regarding the spatial disparities between wild boar habitats and ASF-managed areas, affecting the applicability of their findings.

To address these limitations, we applied ensemble SDMs with two types of datasets to estimate the disparities between wild boar habitats and ASF-managed areas. We estimated wild boar habitats using national-scale survey datasets of wild boar traces and ASF-managed areas, that were confirmed as hunting and carcass elimination locations. We compared the spatial information and quantified the disparity areas using kernel density estimation (KDE) [39]. Ultimately, through our estimation of disparities, we could identify areas where management efforts were lacking, and anticipate a decrease in the uncertainty of ASF management.

## 2. Materials and Methods

### 2.1. Study Area

**Figure 1:**
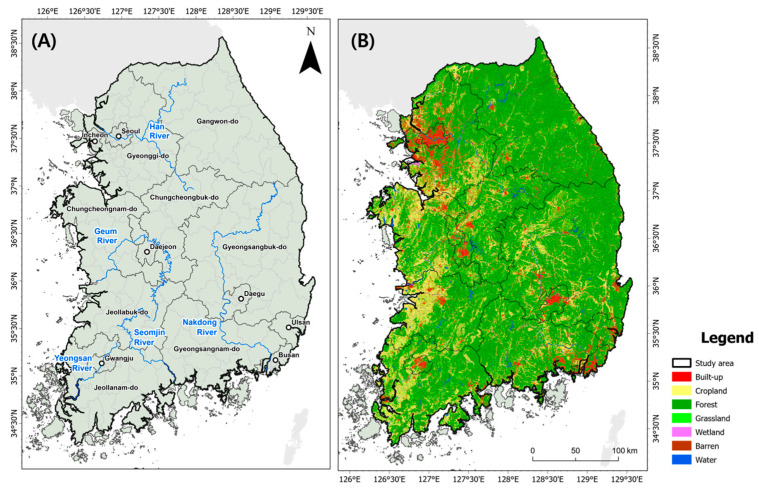
(**A**) Location of main rivers and cities in South Korea; (**B**) Land cover of South Korea.

We set the study area as the entire South Korean territory without islands (34.04°–38.61° N; 125.8°–129.63° E) to estimate the disparities between wild boar habitats and ASF-managed areas (Figure 1). The study area covered 93,442 km^2^ and consisted of forest land (over 60%) predominantly, followed by farmland (18%) and urbanized areas (7%). It was located in a temperate zone with four distinct seasons: the winter season being extremely cold and dry, summer being extremely hot and humid with heavy precipitation during a short rainy period from the end of June to the end of July, and spring and fall having mild and dry conditions. The average minimum and maximum temperatures in August (summer) and January (winter) are 19.7 °C and 26.7 °C, and 3.6 and 6.9 °C, respectively. The annual precipitation is 1306.3 mm, and more than half of the precipitation is concentrated during the summer.

### 2.2. Evaluating Disparity between Wild Boar Habitats and ASF-Managed Areas

To delineate the disparities between wild boar habitats and ASF-managed areas, we employed ensemble modeling that incorporated multiple SDMs to ensure sufficient accuracy. In the simulation of the ensemble SDMs, we used two distinct datasets pertaining to wild boar occurrence, each with specific survey objectives. Both datasets indicated the occurrence of wild boar; however, there were differences in their primary survey purposes.

**Figure 2:**
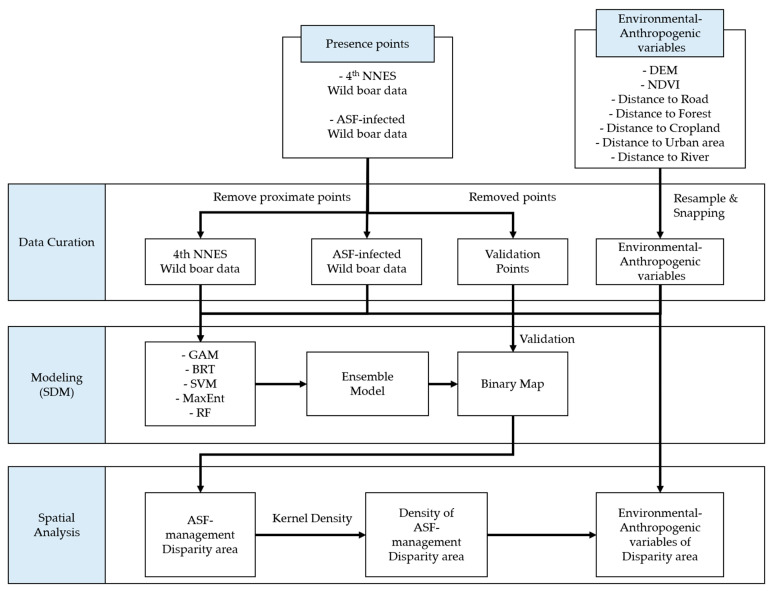
Conceptual images for identifying disparity areas comparing estimation results by wild boar habitats and ASF-managed areas.

The National Natural Environment Surveys (NNESs) dataset encompassed general nationwide traces of wild boar occurrences. Contrarily, the other dataset specifically recorded locations where ASF-infected individuals confirmed hunting practices and carcass collection. Using each of the datasets in the ensemble modeling, one of our results could explain wild boar habitats, whereas the other describes ASF-managed areas. Following the simulation, we conducted a comparative analysis of the ensemble modeling results to evaluate the areas where disparities existed between wild boar habitats and ASF-managed areas (Figure 2).

#### 2.2.1. SDMs

Ensemble SDMs can supplement various SDM techniques by running related SDMs and synthesizing results to improve accuracy [40,41]. Currently, it is widely used to predict species habitats and detect specific patterns in ecosystems [33,42]. In this study, we utilized an ensemble SDM based on five types of SDMs to estimate wild boar habitats and potential ASF-managed areas. We applied the ‘sdm’ R package (version 1.1.8), which supports the generation of multiple SDMs and the ensemble modeling process. We applied SVM, BRT, GAM, RF, and MaxEnt to the ensemble modeling in the package. The estimated distribution probability by ensemble modeling was based on calculating the average of 50 models generated by 10 replicates in five SDMs. The following descriptions explain the SDMs applied to ensemble modeling:

- SVM, originally developed by Cortes and Vapnik (1995) [43], is an inductive modeling technique primarily used for data processing. This algorithm determines the maximum margin hyperplane that represents the greatest separation between classes. Therefore, the SVM is placed close to the decision boundary, and it is very useful to avoid overfitting, yielding an excellent generalization performance for solving numerous non-linear regression and time-series problems, and requires only minimal model tuning with a small training dataset.
- The BRT model has been one of the most widely used approaches over the past two decades [44]. It is adept at handling nonlinearity, selecting predictor variables, and quantifying the relative importance of predictors for ecological questions. BRT outperformed regression-based models in analyzing complex species core-habitat relationships [44,45,46].
- GAM, originally developed by Hastie and Tibshirani (2017) [47], is a general linear model in which the linear response variable depends linearly on unknown smooth functions using predictor variables. It was used to predict relative abundance based on the species occurrence dataset. GAM is to concentrate on constructing one “best model”, which has a specialty than other SDMs. GAM is also used to predict species habitats in a variety of ecosystems and occasionally to detect specific ecological events.
- RF, originally developed by Ho (1995) [48], is method for classification and regression that is conducted by constructing multiple decision trees during model training. In classification (or prediction), the results are created using the average prediction from the individual tree returns. This method can handle both linear and nonlinear relationships and prevent overfitting in the training set. However, this method is difficult to interpret because of its complexity. Additionally, it is computationally intensive for large datasets.
- MaxEnt, originally developed by Phillips et al. (2006) [49], is a well-known SDM technique. This is called maximum entropy modeling and is based on the density estimation principle of Jaynes (1957) [50,51]. Based on the variables (topographic, climatic, anthropogenic, etc.) and occurrence locations, the model can distribute a probability that represents the suitability of the conditions for the target species. MaxEnt was implemented in a presence-only analysis; thus, it did not rely on the confirmed absence data.

#### 2.2.2. Data Curation

##### Point Datasets

We employed two distinct sets of point datasets to estimate wild boar habitats and ASF-managed areas. The first dataset originated from the NNESs, a nationwide initiative conducted by the Korean Ministry of Environment, spanning 1997 to 2019, with updates, every five years. Our analysis specifically leveraged data from the 4th NNES conducted between 2014 and 2018, which consisted of 3665 confirmed wild boar traces (Figure S1). The second dataset comprised locations of confirmed ASF-infected wild boars provided by the Korean Ministry of Environment [52]. This dataset encompassed the period from the initial outbreak of ASF in South Korea, from September 2019 to August 2023, and consisted of 2552 infected wild boars (Figure S2).

ASF infection in wild boars was documented on 16 September 2019, in the northern region of Gyeonggi-do. Subsequently, the contagion expanded into northern Gangwon-do in January 2020, followed by further dissemination into Chungcheongbuk-do in November 2021 and Gyeongsangbuk-do in February 2022. Notably, the detection of infected wild boars exhibited a distinct temporal pattern, with pronounced spikes during the breeding season from November to January, peaking from February to April, and subsequently experiencing a significant decline from June to September [38,53].

**Figure 3:**
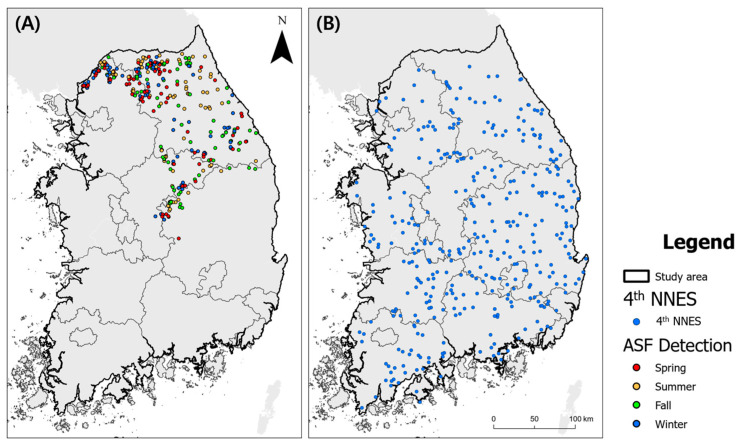
The datasets applied in the study. (**A**) The 360 locations where the ASF-infected individuals were confirmed, consisting of 360 points. (**B**) The 360 locations where wild boar traces were detected, generated from the 4th NNES points.

To ensure the robustness of our datasets, we selected 360 points from each dataset and removed temporally or spatially proximate points to mitigate potential data bias. In the pre-processing of the NNES dataset, we employed a 5 km grid system in our study area and excluded points falling within the same grid cell (Figure 3) [54]. We extracted 30 ASF-infected wild boar points per season from September 2019 to December 2022. This selection process aimed to enhance the representativeness and integrity of our dataset for subsequent analysis.

##### Environmental Variables

**Figure 4:**
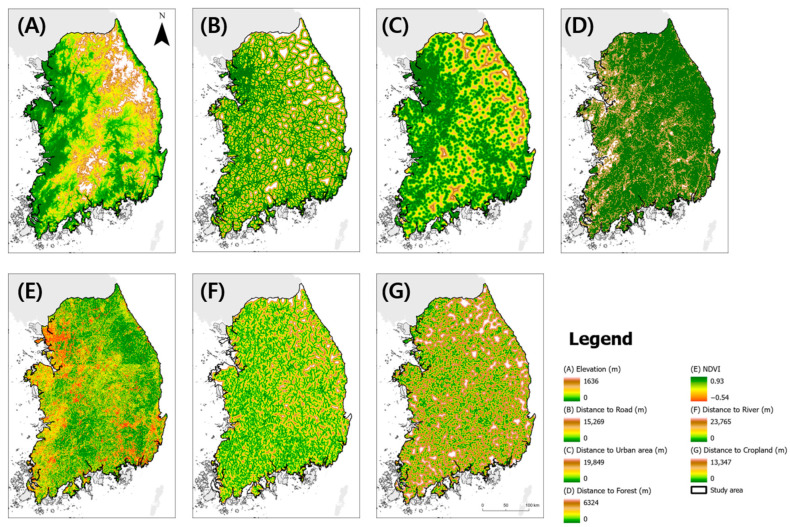
The environmental-anthropogenic variables used to estimate the wild boar habitats and ASF-managed areas.

We curated a set of seven variables, including elevation, distance to road, distance to river, distance to forest, distance to urban areas, distance to cropland, and vegetation index, which were selected based on their anticipated influence on the selection of potential wild boar habitats and ASF-managed areas (Figure 4) [37,55,56]. All the environmental and anthropogenic variables were transformed into raster data at a consistent resolution of 1 km. Forest, cropland, and urban areas -specifically forest, cropland, and built-up cover - were extracted from the European Space Agency (ESA) WorldCoverV200 dataset. These datasets serve as essential proxies for land cover characteristics. The following descriptions were used to derive the environmental variables:

- Elevation: To ensure uniformity, we resampled the elevation data to a 1 km resolution using the Resample tool in ArcGIS pro 3.1.0. These elevation data were sourced from a 30 m resolution Digital Elevation Model (DEM) provided by the National Geographic Information Institute.
- Road Network: Road network data were sourced from Geofabrik OpenStreetMap, which encompasses major roads classified as primary, secondary, and trunk roads. These data aid in understanding the accessibility and connectivity of landscapes.
- River Network: The river network was derived from a river network map provided by the Korean Ministry of Environment, which offered insights into the proximity and distribution of water bodies.
- Normalized Difference Vegetation Index (NDVI): Using the Google Earth Engine, we obtained the available Sentinel-2 surface reflectance imagery from 2020 to 2022 and created a composite image. We calculated the NDVI using imagery captured from June to September, coinciding with peak dietary intensity. The NDVI values were subsequently averaged to indicate the vegetation health and density during this critical period.
- Using the Euclidean distance tool in ArcGIS Pro 3.1.0, we calculated the distance of each raster cell to the nearest forest, urban area, road, cropland, and river using the ESA WorldCoverV200. These variables provide valuable insights into the proximity of the key environmental features in each cell.

The selected environmental variables were transformed into a consistent raster format and served as pivotal inputs for our predictive model, offering a comprehensive understanding of the landscape factors influencing wild boar habitat selection and ASF management patterns.

#### 2.2.3. Ensemble SDM Simulation

##### The Suitability of Wild Boar Habitats and ASF-Managed Areas

As this study only dealt with occurrence data, we randomly generated 360 pseudo-absence points for each model. We used the bootstrap method for validation, partitioning 70% of the data for training and 30% for testing. All the other model settings were identical [57].

Contrary to the NNES points, which offer a broader spatial coverage, ASF infections continued to expand in South Korea. Therefore, the ASF-infected individuals were primarily clustered in northern Gyeonggi-do, Gangwon-do, Chungcheongbuk-do, and Gyeongsangbuk-do. However, spatially biased occurrence data obtained by clustering are limited in terms of estimation and validation [58]. To overcome the problems associated with data clustering, we confined our training areas to north of 36.26°latitude, where the ASF has been documented to have spread. Following the simulation, we generated and averaged the area under the receiver operating characteristic curve (ROC-AUC) to validate the suitability of wild boar habitats and ASF-managed areas (Figure S3). The AUC values are widely recognized metrics for validating the performance of SDMs.

##### Estimation of Wild Boar Habitats and ASF-Managed Areas

To facilitate a comparative analysis of the suitability of wild boar habitats and ASF-managed areas, an ensemble model was synthesized and subsequently converted into a binary map by applying defined thresholds. These ensemble models were built by averaging the output values derived from 50 models generated from each dataset. The threshold for converting the ensemble model into a binary map was determined as the average threshold estimated from 50 individual models [59]. The selection of the optimal threshold for each model was guided by maximizing the sum of the sensitivity (proportion of correctly predicted presence points) and specificity (proportion of correctly predicted absences) (hereafter maxSSS) criteria [60]. MaxSSS is the most valid thresholding method when presence-only data are available [61].

Our methodology estimated the probability maps originating from both modeling of wild boar habitats and ASF-managed areas into binary maps using established thresholds. In this binary representation, areas featuring wild boar habitats and ASF-managed areas were denoted by 1, whereas regions lacking these characteristics received a value of 0.

To assess our estimated binary maps, we applied additional presence point datasets (Figures S1 and S2) based on wild boar traces and confirmed the location of ASF-infected individuals. These point datasets excluded the 360 points (Figure 3) that we used to estimate the suitability of wild boar habitats and ASF-managed areas, comprising 3305 points for wild boar traces and 2192 for confirmed locations of ASF-infected individuals. In the assessment process, we used only precision because of the lack of absence-point datasets.

#### 2.2.4. Evaluating Disparity Areas

Subsequently, we conducted a comparative analysis of the overlying regions evaluated as wild boar habitats and areas that were not classified as ASF-managed areas. This process enabled the evaluation of discrepancies in ASF management. We evaluated the disparities between the two binary maps and categorized them into three distinct groups: areas indicating a mismatch in ASF management (labeled 1), areas reflecting an appropriate level of ASF management (labeled 0), and areas indicating an excessive level of ASF management (labeled −1).

Recognizing the critical implications of false negatives in the context of diseases such as ASF, we selectively extracted only the cells that were assigned a value of 1. These cells were subsequently transformed into point data, and their spatial densities were quantified using the kernel density tool in ArcGIS Pro 3.1.3. KDE is a nonparametric tool used to estimate continuous density distributions [39]. This tool requires the selection of specific parameters, such as search radius and cell size, to ensure precise estimation. In this study, we set the search radius to 10 km, which is the same as the ASF forecasting radius according to the Korean ASF Standard Operating Procedure, and the cell size to 1 km, which is similar to other raster data [62]. Further, we uniformly categorized the density map into five levels (very low, low, moderate, high, and very high) to identify the high-density areas of ASF management disparity.

## 3. Results

### 3.1. The Suitability of Wild Boar Habitats and ASF-Managed Areas

**Figure 5:**
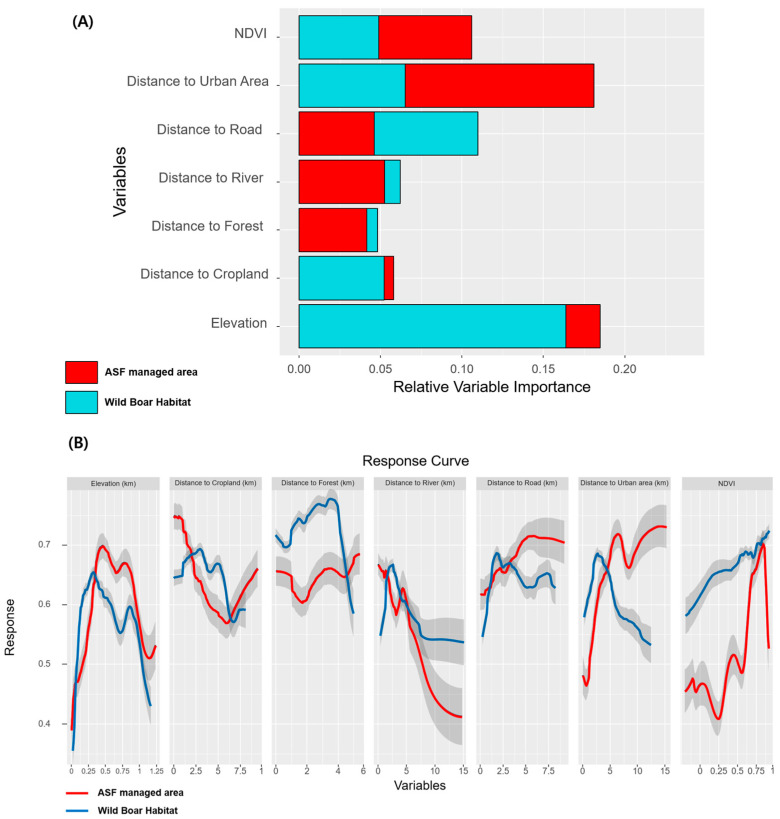
Mean relative variable importance (**A**) and response curves (**B**) of the environmental variables for SDMs.

In the present study, we employed five SDMs to estimate the suitability of wild boar habitats and ASF-managed areas. Our estimates were assessed using ROC-AUC curve. Among the models used to predict wild boar habitat suitability, the RF model exhibited the highest accuracy with a ROC-AUC value of 0.89. In comparison, the SVM and BRT models achieved ROC-AUC values of 0.76 and 0.75, respectively (Table 1). Figure 5A shows the influence of various environmental variables. The main contributors to wild boar habitat suitability were elevation and distance to roads.

Particularly, elevation exhibited a noteworthy response curve (Figure 5B), indicating a rapid increase in suitability up to an elevation of 150 m, with a peak at 300 m. The response curve for distance to roads revealed a similar influence, reaching its zenith at 2500 m, after which suitability gradually declined with decreasing.

Among the models estimating ASF-managed area suitability, the RF model exhibited the best performance, yielding the highest ROC-AUC value of 0.92. Following behind were the BRT and SVM models with ROC-AUC values of 0.79 and 0.77, respectively. Influential variables in estimating ASF-managed area suitability, such as elevation and distance to urban areas, were noted crucial factors. The response curve for elevation concerning ASF-managed area suitability exhibited a relatively gradual increase compared with its counterpart for wild boar habitat suitability, culminating at 500 m. The response curve for the distance to urban areas displayed a sharp increase at greater distances from urban areas.

Notably, NDVI and distance to forest, two less influential variables, displayed distinct response curve patterns. The estimation of wild boar habitat suitability revealed an increase in suitability in areas farther from forests, with higher NDVI values. However, the distance to the forest variable did not exhibit significant patterns while estimating the suitability of ASF-managed areas. Furthermore, NDVI influenced the estimation of ASF-managed area suitability at the highest values.

### 3.2. Estimation of Wild Boar Habitats and ASF-Managed Areas

**Figure 6:**
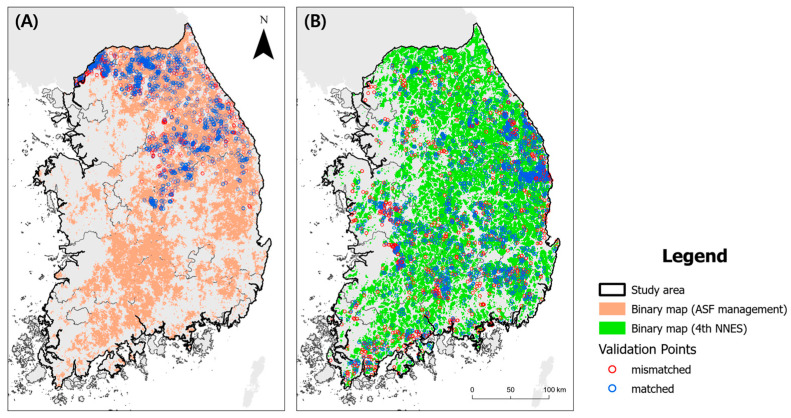
Binary map and validation points of ASF management model (**A**) and wild boar habitat model (**B**).

We distinguished the cells as wild boar habitats and ASF-managed areas using maxSSS, which was used to determine the threshold for evaluating the binary maps. The thresholds for wild boar habitat and ASF-managed area suitability were 0.51 and 0.48, respectively. We assigned areas above this threshold as wild boar habitats (ASF-managed areas). Conversely, areas below this threshold were assigned as non-wild boar habitats (or non-ASF-managed areas) (Figure 6).

The results of the binary map assessment showed a precision of 74% in the comparison between wild boar habitat estimation and wild boar trace point data. The 2443 out of the 3305 points were located within wild boar habitats. The precision was 74% in the comparison between the ASF-managed area estimation and the confirmed location of ASF-infected individuals. A total of 1626 of the 2192 points were located within ASF-managed areas.

Approximately 48% of the total study area in the binary map, representing wild boar habitats, exceeded the predetermined threshold. Forest regions within the study area were predominantly categorized as wild boar habitats, whereas most areas in close proximity to urban areas and roads did not meet the threshold criteria. A binary map designed to identify the ASF-managed areas revealed that 41% of thestudy area exceeded this threshold. ASF-managed areas are predominantly situated within forested region sand, exhibit a pattern reminiscent of wild boar habitats. However, substantial areas with low probability values were observed around urban areas like Cheongju and Daejeon cities, as well as in locations such as Mungyeong-si, Daegu-si, Pohang-si, and Gyeongju-si.

### 3.3. Evaluating Disparity Areas

**Figure 7:**
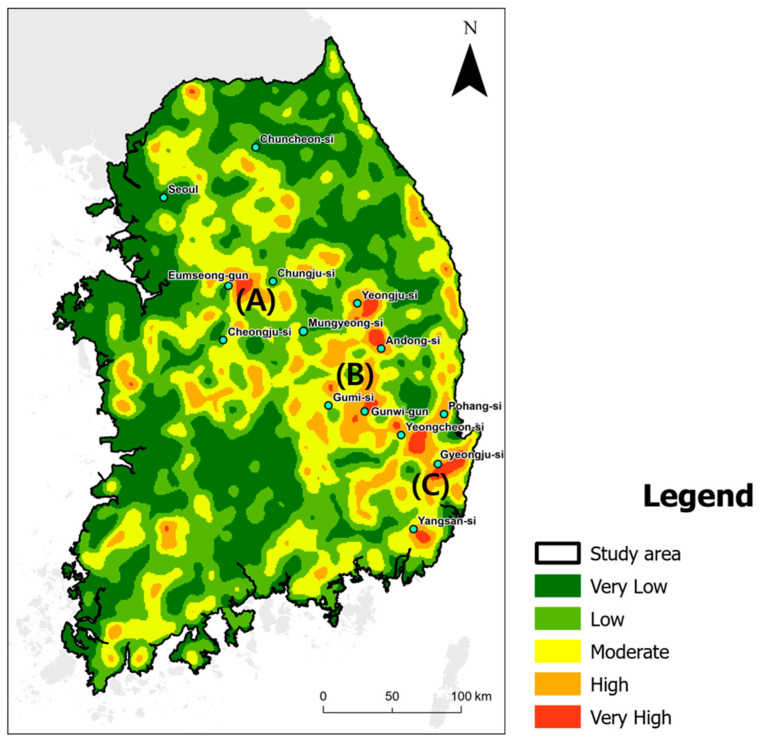
Density of ASF-management disparity areas. Eumseong-gun in Chungcheongbuk-do Province (A), Yeongju-si in Gyeongsangbuk-do Province (B), and mountainous expanse extending from Yeongcheon-si in Gyeongsangbuk-do Province to Gyeongju-si in Gyeongsangnam-do Province (C) were evaluated as having high disparities.

To evaluate the disparities between potential wild boar habitats and ASF-managed areas, and to estimate the areas where these disparities were concentrated creating a potential risk for ASF transmission, we conducted a comparative analysis of two binary maps. The KDE analysis resulted in a density map with values ranging from 0~0.43, and we classified these values into five levels (very low: 0~0.1, low: 0.1~0.2, moderate: 0.2~0.3, high: 0.3~0.4, very high: 0.4~0.5) (Figure 7). The observed disparities were not uniformly distributed across the country, rather exhibited sporadic distribution patterns. Dense forests and intensely developed regions, such as Seoul, Chuncheon-si, Chungju-si, and Cheongju-si were evaluated as areas of very low to moderate disparity. The areas in which the urban and forest regions were mixed were evaluated as having high or and very high disparities, such as Eumseong-gun in Chungcheongbuk-do Province (designated as A), Yeongju-si in Gyeongsangbuk-do Province (designated as B), and the mountainous expanse extending from Yeongcheon-si in Gyeongsangbuk-do Province to Gyeongju-si in Gyeongsangnam-do Province (designated as C).

## 4. Discussion

ASF transmission has significantly damaged global food and economic security, and humans have attempted to prevent ASF transmission by hunting and installing barriers [19,20,21]. However, ASF is continuously increasing and being transmitted to other regions [3]. Thus, the ASF-managed areas that prevent ASF transmission have limited coverage of overall wild boar habitats.

In this study, we built ensemble models to estimate the suitability of wild boar habitats and ASF-managed areas in South Korea, so as to evaluate the disparities in ASF management (Figure 7). Our models showed prominent performance, with an ROC-AUC of 0.76 in the estimation of wild boar habitats and 0.79 in ASF-managed areas (Table 1). The response curve patterns of the significant environmental variables in both estimations were similar, except for the distance to forest and NDVI (Figure 5B). Therefore, ASF management is generally well practiced in South Korea. However, differences in variable contributions and response curves between the two ensemble models suggest differences in potential wild boar habitats and ASF management strategies.

This study identified distance to forest and NDVI as predictive variables for management disparities, with wild boar habitat having a higher response than ASF-managed areas. The suitability of wild boar habitats increased continuously in dense forests with high NDVI (Figure 5B). However, ASF-managed areas showed no significant change in response curves at distances 0 to 4 km from forest and NDVI scores below 0.6, which was lower than the response curve for wild boar. This indicates that wild boars prefer dense forests as core habitats, whereas ASF management cannot be implemented in some regions in dense forests near urban areas. Although ASF management has been implemented in extensive wild boar habitats of South Korea, some minor regions with dense forests have a high risk of ASF transmission [16,63,64].

Our evaluation of the disparities generated from the comparison between the binary maps of wild boar habitats and ASF-managed areas showed a high precision value (0.74). These disparities reveal potentially less implemented areas of ASF management. The differences in the response curves with fewer contributing variables in the two models were reflected in the characteristics of these regions. Most of the disparities were in dense forests with high NDVI value close to urban areas. Using KDE analysis, we identified a high density of areas with disparities in Eumseong-gun in Chungcheongbuk-do Province; Yeongju-si and Andong-si in Gyeongsangbuk-do Province; and in the region from Guwni-gunto Yangsan-si in Gyeongsangnam-do Province. In particular, the high-density disparity area connecting Yeongju-si to Yangsan-si could be a significant ASF transmission corridor in Gyeongsangnam-do Province. However, further analysis, connectivity analysis, and agent-based modeling are needed to reflect the ecological characteristics of wild boars and the behavior of managers, and to determine if areas with high ASF management inconsistencies are turning into dispersal paths. Further analysis to determine whether changes in the direction of wild boar population movement due to hunting and fencing should also be considered [65,66,67].

## 5. Conclusions

Efforts to manage ASF by hunting wild boar and removing carcasses from forests are costly and time-consuming. To enhance management efficiency, it is essential to establish precise search zones that consider the ecological attributes of wild boar. In the context of this study, our analysis focused on identifying the existing disparities between ASF management strategies and the habitats of wild boars. We identified specific regions where these disparities were particularly pronounced. These findings hold significant potential for identifying areas where ASF control is lacking and the environmental characteristics of these areas to refine management strategies and establish proactive control measures. By reducing the disparities between ASF control efforts and habitat preferences of wild boars, we aimed to optimize the allocation of resources and maximize the effectiveness of disease management initiatives.

## Figures and Tables

**Table 1:** ROC-AUC values in the simulation of each model.

	GAM	BRT	SVM	MaxEnt	RF	Ensemble
Wild boar habitat	0.71	0.75	0.76	0.72	0.89	0.76
ASF-managed area	0.75	0.79	0.77	0.75	0.92	0.79

## Data Availability

Data are contained within the article and Supplementary Materials.

## Supplementary Materials

The following supporting information can be downloaded at: https://www.mdpi.com/article/10.3390/ani13223482/s1, Figure S1: The spatial distribution of wild boar trace points from 4th National Natural Environment Survey. The points were used to predict (red) and assess (blue) potential wild boar habitats; Figure S2: The spatial distribution of ASF-infected wild boar points provided by the Korean Ministry of Environment. The points were used to predict (red) and assess (blue) potential ASF-managed areas; Figure S3: The results of ASF-managed areas (A) and wild boar habitats (B) ensemble species distribution model.
