# Supplementary material for: Predicting Disparity between ASF-Managed Areas and Wild Boar Habitats: A Case of South Korea
Source: Animals (Basel). 2023 Nov 11;13(22):3482. doi: 10.3390/ani13223482 (PMC10668782; doi:10.3390/ani13223482)

Supplementary Figures & Tables

Figure S1: The spatial distribution of wild boar trace points from 4th National Natural Environment Survey. The points were used to predict (red) and assess (blue) potential wild boar habitats.

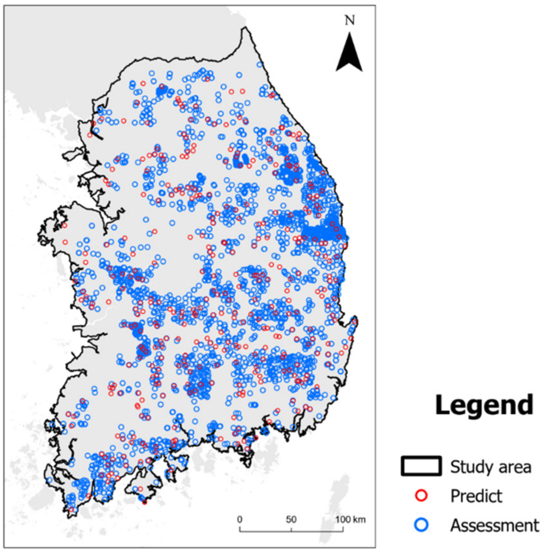

Figure S2: The spatial distribution of ASF-infected wild boar points provided by the Korean Ministry of Environment. The points were used to predict (red) and assess (blue) potential ASF-managed areas.

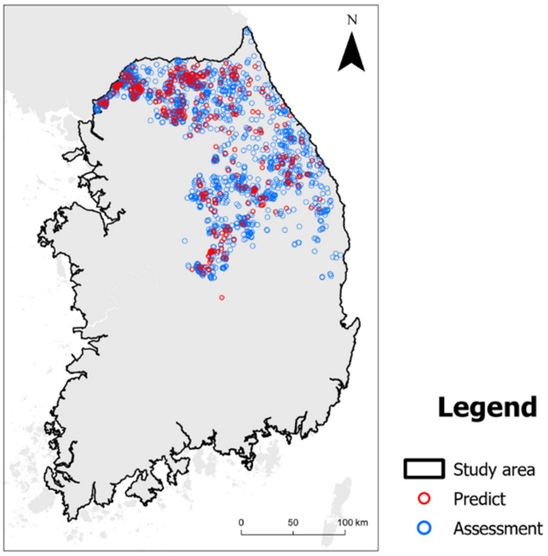

Figure S3: The results of ASF-managed areas (A) and wild boar habitats (B) ensemble species distribution model.

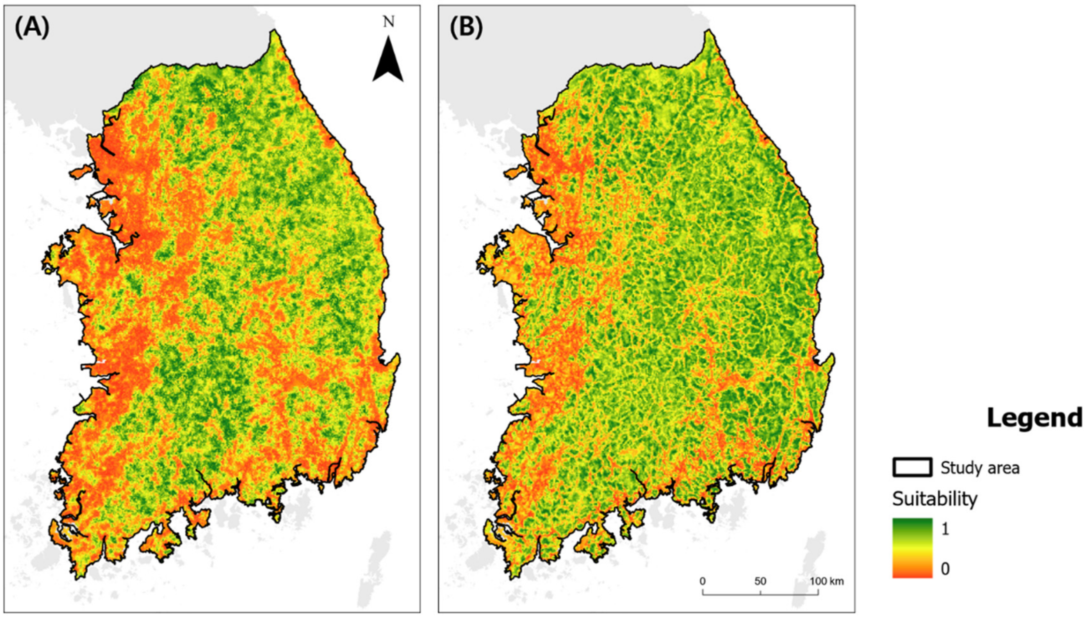

Supplement: Supplementary file 1 [file animals-13-03482-s001.zip › animals-2659757-supplementary.pdf]
